# High-precision CA-ID-TIMS zircon U-Pb geochronology: a review of the Neoproterozoic time scale

**DOI:** 10.1093/nsr/nwaf206

**Published:** 2025-05-21

**Authors:** Chuan Yang, Fred Bowyer, Daniel Condon

**Affiliations:** State Key Laboratory of Palaeobiology and Stratigraphy, Nanjing Institute of Geology and Palaeontology, Chinese Academy of Sciences, Nanjing 210008, China; School of Earth and Environment, University of Leeds, Leeds LS2 9JT, UK; Geochronology and Tracers Facility, British Geological Survey, Keyworth NG12 5GG, UK

**Keywords:** Neoproterozoic, zircon U-Pb, ID-TIMS, carbon isotope excursion, Snowball Earth

## Abstract

The Neoproterozoic Era is a critical time interval when the Earth experienced fundamental changes, manifested as Snowball Earth climatic extremes, large fluctuations in oceanic and atmospheric compositions, and emergence and rapid diversification of animals. High-precision geochronology of Neoproterozoic stratigraphy is essential for constraining timings, durations, and rates of these major events, and for assessing the synchroneity and nature of interactions between them. Here we review recent advances in the CA-ID-TIMS zircon U-Pb dating method and discuss the factors that influence the choice of method used to date Neoproterozoic stratigraphy. Advances in the temporal calibration of major carbonate carbon isotope excursions, glaciations, and fossil assemblages of the Neoproterozoic using high-precision age constraints, are also reviewed. This enables us to construct a composite carbonate carbon isotope profile for the Neoproterozoic, which is anchored by radio-isotopic ages. Together with available biodiversity metrics, this provides critical insights into the co-evolution of life and environment in the Neoproterozoic.

## INTRODUCTION

The Neoproterozoic Era [1000 to 538.8 million years ago (Ma), the International Chronostratigraphic Chart 2024] was a critical time interval when the Earth experienced fundamental changes in environment and life evolution. Correlation and integration of disparate stratigraphic sections are crucial to understanding the nature and causes of glaciations, large perturbations of biogeochemical cycles, and key evolutionary innovations that took place during the Neoproterozoic. Development of a Neoproterozoic temporal framework of sufficient resolution has been increasingly dependent on absolute age constraints from radio-isotopic dating, combined with relative chronologies derived from stratigraphic relationships and astrochronology [1–5].

A detailed review was published more than a decade ago that described various radio-isotopic techniques used for dating of Neoproterozoic stratigraphy [6]. This present contribution will mainly focus on the high-precision U-Pb geochronometer and provides an overview of advances in the CA-ID-TIMS (Chemical Abrasion-Isotope Dilution-Thermal Ionization Mass Spectrometry) zircon U-Pb dating method used for Neoproterozoic geochronology. The Re-Os geochronometer has also been applied to many organic-rich Neoproterozoic stratigraphic intervals and has become an important method to date sedimentary sequences devoid of volcanogenic rocks [7,8]. For a recent review of the Re-Os geochronometer, see Li *et al.* [9] in this issue. In this contribution we will also discuss specific examples where a better understanding of the nature and inter-relationships of Neoproterozoic events has been achieved through increasing availability of precise radio-isotopic ages over the past decade.

## ADVANCES IN THE CA-ID-TIMS ZIRCON U-PB METHOD

Methodological advances in CA-ID-TIMS zircon U-Pb geochronology over the past decade include low total procedural blank, a better understanding of chemical abrasion, precisely calibrated U-Pb tracers, advanced mass spectrometry, and more informed age interpretation [10].

**Figure 1.**
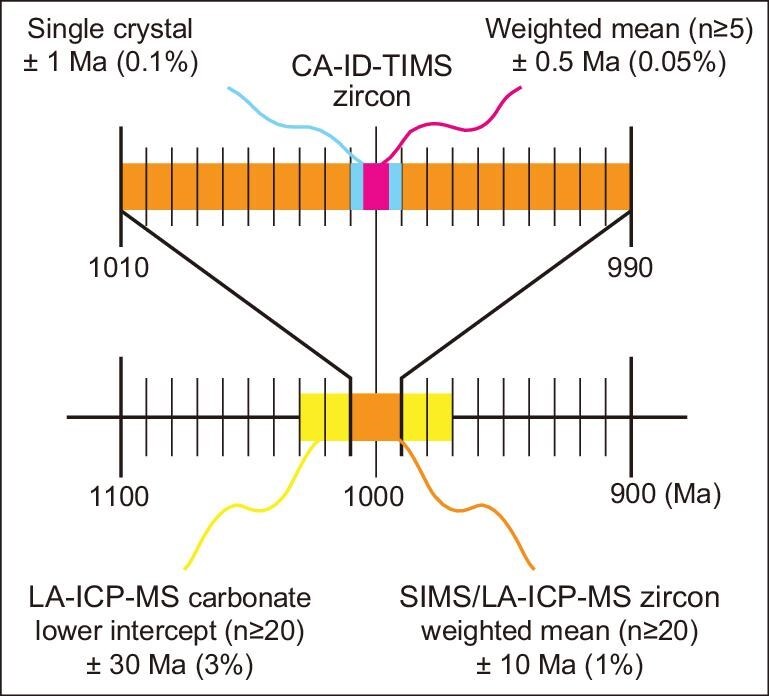
Representative analytical precision (2σ) of dating results obtained by LA-ICP-MS carbonate, SIMS/LA-ICP-MS zircon, and CA-ID-TIMS zircon U-Pb methods. Modified after [38].

Since the late 20th century, the development of clean lab facilities, the use of ultra-clean reagents and Teflon products, standardized operation procedures, and global phase-out of leaded gasoline have significantly reduced the procedural Pb blank of the ID-TIMS zircon U-Pb method. Continued efforts in each of these aspects during the past decade have permitted very low procedural Pb blank at a level of ≤0.2 pg [11,12], which enables zircon grains with moderate (≥10 pg) radiogenic Pb to be dated with a precision of 0.1% on the U-Pb ratio for single-grain analyses (Fig. 1). These advances have also facilitated a trend towards decreasing sample size in CA-ID-TIMS zircon U-Pb geochronology, permitting high-precision dating of small zircon fractions obtained by micro-sampling techniques such as laser and focused ion beam and therefore adding spatial resolution to high-precision dates [13,14].

Partial loss of radiogenic Pb is one of the main obstacles to the accuracy and precision of zircon U-Pb geochronology. Air abrasion was developed in the last century to minimize the effect of Pb loss via mechanically removing the outer parts of zircon grains [15]. To tackle Pb loss that might also affect grain interiors, the chemical abrasion approach was established in 2005 [16] and has been continually refined in the past decade [17–19]. This approach combines a high-temperature annealing step followed by a partial dissolution in hydrofluoric acid to remove damaged parts of the zircon crystals that underwent Pb loss. The most commonly used or recommended temperatures and durations in zircon chemical abrasion include 180°C for 12 h, 190°C for 15 h [18], 210°C for 8 h [20], and 210°C for 12 h [19]. Recently, microscale X-ray computed tomography, scanning electron microscopy, and Raman spectroscopy were used to evaluate zircon dissolution behavior in HF [17]. These microstructural investigations reveal that the degree of radiation damage, defect distribution, and the size and position of inclusions exert controls on zircon dissolution during chemical abrasion, indicating that the selection of the chemical abrasion condition is sample-dependent [17].

**Figure 2.**
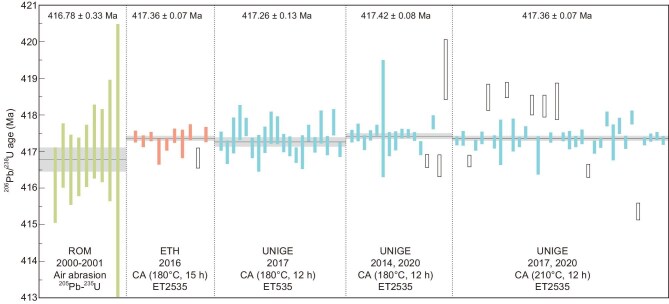
Comparison of ID-TIMS U-Pb analyses of the zircon standard Temora 2. Vertical bar lengths represent 2σ analytical uncertainty of individual analyses; open bars are excluded from weighted mean date calculation following the original interpretations. Horizontal lines signify calculated sample dates and the width of the shaded band represents internal uncertainty in the weighted mean date at 2σ level. The Royal Ontario Museum (ROM) data set is from [119]. The ETH Zurich data set is from [28]. And the data sets from the University of Geneva (UNIGE) are from [26]. CA = chemical abrasion.

The ID-TIMS U-Pb method requires isotopically enriched U-Pb tracers to determine the concentration and isotopic composition of both U and Pb and therefore the U/Pb ratio of the sample. The development of nuclear reaction and isotope separation techniques had significantly facilitated the production of highly enriched U-Pb tracers [21] and the transition of tracers from a single, enriched natural isotope (e.g. ^208^Pb) to double, synthetic isotopes (e.g. ^202^Pb-^205^Pb) in the last century. During the past decade, two U-Pb tracers, ET535 (^205^Pb-^233^U-^235^U) and ET2535 (^202^Pb-^205^Pb-^233^U-^235^U), have been prepared and precisely calibrated with their isotopic compositions traced back to SI units under the auspices of the EARTHTIME Initiative [22,23]. These have since been widely used in high-precision U-Pb geochronology labs worldwide (Fig. 2), effectively eliminating a major source of inter-laboratory bias. The use of a ^233^U-^235^U double spike to correct for mass fractionation requires *a priori* knowledge of the ^238^U/^235^U ratio of the sample [22], a prerequisite that is commonly met when dating zircon grains that have an average ^238^U/^235^U value of 137.818 ± 0.045 (2σ) [24]. This ^238^U/^235^U_zircon_ ratio and uncertainty can be applied to other high-temperature terrestrial dating materials, because of their similar ^238^U/^235^U ratios to that of zircon and limited ^238^U/^235^U variability [24]. The use of U-Pb tracers that include ^233^U-^236^U [18] is preferable when dating materials with unknown ^238^U/^235^U ratios, allowing simultaneous determination of U isotope composition provided enough U is present in the sample and uranium oxide isobaric interferences can be accurately corrected. This can also be achieved by using a ^233^U-^236^U double spike on an aliquot of the sample in conjunction with U-Pb analyses using EARTHTIME U-Pb tracers [22].

While some high-precision U-Pb geochronology labs use MC-ICP-MS (Multicollector-Inductively Coupled Plasma-Mass Spectrometry) to measure U isotopic composition [18,25], most labs measure Pb and U by TIMS. Recent advances in TIMS have facilitated the high-precision measurements of small ion beams so that an unprecedented precision of 0.01% or better for the ID-TIMS ^206^Pb/^238^U date is achievable [26–29]. Newly developed amplifiers for the Thermo Scientific^™^ TIMS (and also MC-ICP-MS), which are equipped with 10^13^ Ω feedback resistors, have a theoretical improvement in signal/noise of 10 times and better reproducibility of baseline and external precision relative to the 10^11^ Ω amplifiers, and yield more precise data compared to secondary electron multiplier for beam sizes higher than 20 kcps [30]. The amplifying system of a Phoenix TIMS, another instrument widely used in the ID-TIMS U-Pb community, has also been updated with the advent of ATONA (‘aA to nA’) amplification technology. The ATONA is a capacitive transimpedance amplifier characterized by low and stable noise, rapid amplifier decay, improved gain stability, and a large dynamic range [31]. The ATONA-Faraday system can provide similar precision to Daly ion counting at average run intensities of 0.5–1 mV, and is anticipated to become superior to the ion counting measurements for ion currents of >1 mV (∼ 60 kcps) [27]. A further advance of the ATONA, the Zeptona detector system, provides even better noise performance and baseline stability and larger dynamic range [32]. These advances in amplification technology have greatly improved the detection limits and noise levels of the Faraday detectors and therefore expanded the applicability of Faraday analyses to pg-sized Pb samples.

A U-Pb age of geological significance is an interpretation of a date or a set of dates under the geological context of the dated sample. This is not straightforward and is usually complicated by date scatter caused by the presence of antecrysts, protracted zircon growth, and residual lead loss [11,33]. In some cases, antecrystic zircon grains can be identified by trace element compositions and Hf-O isotopes; however, distinguishing them from the protracted autocrystic zircon is sometimes subjective [34]. Protracted zircon growth has been revealed to be ubiquitous in magmatic systems [34], and its impediment to age interpretation could be eliminated by the Bayesian Markov Chain Monte Carlo approach which uses theoretical/empirical zircon crystallization distributions as prior information to constrain a likelihood-based Bayesian eruption age [35]. Residual lead loss remains a great challenge to zircon CA-ID-TIMS U-Pb age interpretation even following chemical abrasion. The most commonly used statistical models in ID-TIMS U-Pb geochronology, namely the traditional weighted mean, the youngest single zircon U-Pb date [36], and the Bayesian Markov Chain Monte Carlo approach [35], would all yield younger results if residual lead loss existed. Although zircon dissolution during chemical abrasion is not entirely controlled by radiation damage content [17], quantifying the radiation accumulation in zircon and optimizing chemical abrasion conditions accordingly will mitigate the age interpretation bias caused by residual lead loss. Weighted mean ages that young-upwards and thereby follow the principle of stratigraphic superposition [37], along with increasingly large numbers of single grain analyses (10–20) per ash bed [10], should facilitate accurate interpretations of zircon U-Pb data. In cases where zircon dates do not follow superposition, it may also be necessary to consider uncertainties in stratigraphic correlation and the potential for these data to reveal cryptic structural displacements.

## CHOICE OF METHOD

Based on the U-Pb decay system, many analytical protocols have been employed to obtain absolute ages from minerals and rocks. They yield different levels of precision, with the most widely used methods yielding age uncertainties in the range ∼3% to ≤0.05% (Fig. 1). Specifically, the CA-ID-TIMS zircon U-Pb method yields individual crystal-fragment and weighted mean ^206^Pb/^238^U dates of 0.1% and ≤0.05% relative precision (2σ unless otherwise stated), respectively [38]. However, because of the variability of natural material (protracted growth, the presence of antecrystic or xenocrystic domains, or residual lead loss), repeated analysis of natural reference zircon has not shown reproducibility of its ^206^Pb/^238^U date better than 0.05% [26]. A recent interlaboratory experiment was conducted in which pre-spiked, homogeneous zircon solution was dated using the ID-TIMS method at 11 institutions, and the results demonstrated that the weighted mean ^206^Pb/^238^U dates for the zircon solution agreed within 0.05% (two standard deviations) [39]. Meanwhile, repeated analyses of homogeneous, synthetic EARTHTIME age solutions yield repeatability one order of magnitude better than the natural reference zircon [26]. *In situ* microbeam U-Pb dating methods have higher spatial resolution and are more efficient, but the precision of dating results is much lower than that of the CA-ID-TIMS method (Fig. 1). The precision of SIMS (secondary ion mass spectrometry) and LA-ICP-MS (Laser Ablation-Inductively Coupled Plasma-Mass Spectrometry) zircon U-Pb dating results is usually 1%–2% [40,41], and the LA-ICP-MS U-Pb dates of carbonate typically exceed 3% precision [42].

**Figure 3.**
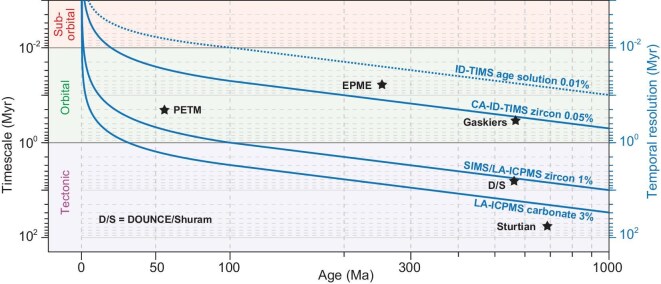
Temporal resolution of U-Pb dating methods as a function of absolute age younger than 1000 Ma. Notice the change of scale at 100 Ma. The underlying mechanism responsible for driving a geochemical perturbation or biotic event can only be resolved by dating methods whose temporal resolution curves are above the timescale of the event on the plot. The precision (0.01%, dashed blue line) of ID-TIMS analysis of the EARTHTIME age solutions is plotted to mark the current upper precision limit of ID-TIMS zircon U-Pb analysis. Tectonic, orbital, and sub-orbital timescales of paleoclimate changes are presented for comparison. PETM = Paleocene-Eocene Thermal Maximum; EPME = End-Permian mass extinction; DOUNCE = Doushantuo negative carbon isotope excursion.

The choice of radio-isotopic dating method mainly relies on the availability of datable material and the timescale of geological process to be resolved, which ranges from tens of millions of years to sub-million years. The uncertainties of each radio-isotopic date will be propagated into the duration (and therefore the rate) calculated from the dates. The implication is that, only when the absolute uncertainties of radio-isotopic dates are much lower than the duration, can we resolve the process/mechanism responsible for driving an event (e.g. volcanic episode driving climatic change leading to oceanic geochemical perturbation). For example, the ∼570 Ma DOUNCE/Shuram carbonate carbon isotope event lasted for ∼6Myr according to cyclostratigraphic estimates [43]. SIMS/LA-ICP-MS zircon U-Pb dating can give us a rough idea about the timing of this event. However, a meaningful duration of the event can only be achieved by high-precision dating approaches such as the CA-ID-TIMS zircon U-Pb method (Fig. 3). In order to disentangle the temporal relationships between short-duration events such as large igneous provinces (LIPs) and mass extinctions, and to temporally calibrate Phanerozoic chronostratigraphic boundaries, high-precision dating approaches should be prioritized.

## DATING THE NEOPROTEROZOIC

### Neoproterozoic carbonate carbon isotope excursions

Interpreting long-term changes in the global carbon cycle from the carbon isotopic composition of marine carbonates (δ^13^C_carb_) can be complicated by syn-to-post depositional over-printing of local effects including diurnal coupling between photosynthesis and carbonate saturation in shallow carbonate settings [44], local pools of dissolved inorganic carbon (DIC) with distinct isotopic compositions [45], and facies-specific and porosity- and permeability-specific degrees and styles of diagenesis [46]. Nevertheless, long-term secular trends that show gradual unidirectional shifts in δ^13^C_carb_, which are consistently recorded between multiple globally distributed but contemporaneous open-marine settings, are most likely to represent global changes in the carbon cycle that are useful for global chemostratigraphic correlation [47].

**Figure 4.**
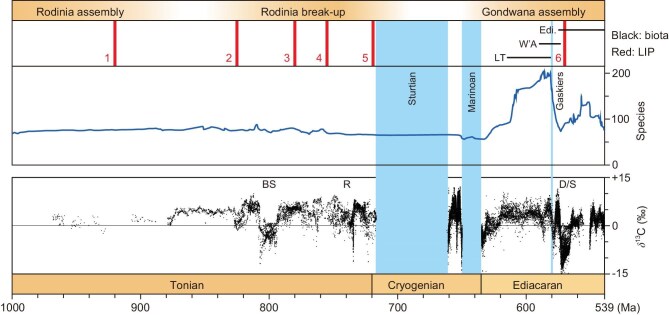
Composite carbonate carbon isotope profile and eukaryotic species richness (including unicellular and multicellular eukaryotes and animals) of the Neoproterozoic with the timings of major glaciations, magmatic episodes, and global tectonic events. The species richness curve is from [110]. The carbonate carbon isotope data are from [1–3,120] and references therein, but see [48] for alternative excursion durations between the pre-Sturtian Bitter Springs and Russøya anomalies and [52] for a longer-duration mid-Cryogenian non-Snowball interval. The timing of LIP events follows [121]. LIP 1 = 920 Ma Dashigou, Bahia-Araquai, and Zadinian-Mayumbian LIPs; LIP 2 = 825 Ma Guibei and Gairdner-Willouran LIPs; LIP 3 = 780 Ma Kanding and Gunbarrel LIPs; LIP 4 = 755 Ma Mundine Well-Keene and Shaba LIPs; LIP 5 = 719 Ma Franklin, Irkutsk, and Hubei–Shaanxi LIPs; LIP 6 = 570 Ma Central Iapetus Magmatic Province. BS = Bitter Springs; R = Russøya; D/S = DOUNCE/Shuram; LT = Lantian biota; W'A = Weng'an biota; Edi. = Ediacara biota.

The Neoproterozoic is marked by perturbations to the global carbon cycle, as evidenced by extreme fluctuations in carbonate carbon isotopes [1–4,48,49]. Carbonate carbon isotope excursions (CIEs) in the Neoproterozoic serve as chemostratigraphic markers for global correlation and data integration, and may also serve as meaningful proxies for global paleo-environmental change associated with perturbations to the climate-carbon cycle. Therefore, a globally-representative Neoproterozoic carbon isotope profile, especially across large-magnitude CIEs, needs to be calibrated by high-precision radio-isotopic dates. An age-calibrated Neoproterozoic carbon isotope profile is presented in Fig. 4. The age constraints on the Bitter Springs CIE, DOUNCE (Doushantuo negative carbon isotope excursion)/Shuram, and BACE (Basal Cambrian negative carbon isotope excursion), which are the most intensively dated chemostratigraphic markers in the Neoproterozoic, are discussed further below. Other Neoproterozoic chemostratigraphic markers such as the ∼735 Ma Russøya [50] and ∼650 Ma Trezona [51,52] anomalies still require future high-precision dating via zircon U-Pb methods and are not discussed in detail here. Unless otherwise stated, the zircon U-Pb dates discussed below are derived from the CA-ID-TIMS U-Pb dating method and their errors are presented in 2-sigma as analytical uncertainty (Supplementary Material).

The Bitter Springs CIE is a negative carbon isotope anomaly (Fig. 4) named after the Bitter Springs Formation of Central Australia where it was first well documented [53]. A large-magnitude negative carbon isotope excursion recorded in the upper Fifteenmile Group, NW Canada, and a similar one in the lower Tambien Group, Ethiopia, have been regarded as equivalents to the Bitter Springs CIE [54,55]. If confirmed, the ash bed zircon U-Pb date of 811.51 ± 0.25 Ma [54] and the black shale Re-Os date of 810.7 ± 6.3 Ma [56] both from horizons below the negative CIE interval in the upper Fifteenmile Group provide maximum age constraints (i.e. the oldest permissible age of the event of interest) on the Bitter Springs CIE, and the ash bed zircon U-Pb date of 788.72 ± 0.24 Ma from a horizon above the negative CIE interval in the lower Tambien Group provides a minimum age constraint [55]. These radio-isotopic dates suggest that the duration of the Bitter Springs CIE is less than ∼23Myr, provided it is a globally correlative event. A 1D thermal subsidence Bayesian age-depth model for the Svalbard composite succession, which is underpinned by global correlation of chemostratigraphic and radio-isotopic data, yields refined timings for the onset and termination of the Bitter Springs CIE of 808.7 + 3.3/−3.5 Ma and 801.9 + 3.2/−3.3 Ma, respectively, resulting in a duration of 6.9 ± 0.2 Myr [48]. Nevertheless, more high-precision radio-isotopic dates are needed to test and refine the timing and duration of the Bitter Springs CIE, given the lack of direct radio-isotopic age constraints on the CIEs from Australia and Svalbard, the uncertainty in global stratigraphic correlation, and the duration of depositional hiatuses represented by unconformities.

The late Ediacaran DOUNCE/Shuram is the largest negative CIE recorded from marine carbonates in the geologic record [57]. The timing of recovery from the DOUNCE in China was previously thought to be constrained by an ash bed zircon U-Pb date of 550.14 ± 0.16 Ma from the top of Miaohe Member, Doushantuo Formation in the Yangtze Gorges area (intra-platform setting) [2,58,59]. However, the correlation between this U-Pb date and the DOUNCE has aroused heated debate, sparked by the regional stratigraphic complexity of the Doushantuo and Dengying formations in the Yangtze Gorges area [60,61]. An ash bed within the stratigraphic interval above a prominent negative CIE in southeastern Guizhou Province (slope setting) of China is dated at 556.38 ± 0.14 Ma [2]. This negative CIE is regarded as an equivalent to the DOUNCE and therefore the date of 556.38 ± 0.14 Ma is interpreted as its minimum age [2], which is further supported by the black shale Re-Os dates of 567.3 ± 3.0 Ma, 567.7 ± 7.4 Ma, and 562.7 ± 3.8 Ma from strata above the Shuram CIE in Oman and the Gametrail CIE in NW Canada [7,62]. The maximum age constraints for the onset of the DOUNCE/Shuram include black shale Re-Os dates of 574.0 ± 4.7 Ma and 578.2 ± 5.9 Ma from strata below the Gametrail CIE in NW Canada and below the Shuram CIE in Oman, respectively [7]. The duration of the DOUNCE/Shuram is suggested to be 6.1 ± 0.2 Myr based on cyclostratigraphy of the Doushantuo Formation in the Yangtze Gorges area [43]. Although a general consensus has been reached that the duration of the DOUNCE/Shuram is <10 Myr, its precise onset and terminal timing are yet to be determined by the CA-ID-TIMS zircon U-Pb method, hindering our ability to explore its temporal and causal relationships with Ediacaran biotic turnover events [2].

The BACE is another large-magnitude negative CIE recorded in Ediacaran-Cambrian transitional strata worldwide [63]. The age of the BACE was previously correlated with a radio-isotopically dated negative CIE in the A4 Member of the Ara Group, Oman at 541.00 ± 0.13 Ma [64]. However, the Ara Group was deposited in an evaporitic basin where depositional hiatuses are likely and the complex stratigraphic relationships of carbonate stringers result in significant lithostratigraphic correlation uncertainty. The age of 541 Ma is derived from an ash bed immediately below carbonates that record a negative CIE in core, and thus should be considered as a maximum age for onset of this CIE [64,65]. A maximum deposition age (i.e. the oldest permissible depositional age of the stratigraphic horizon of interest) of 539.40 ± 0.23 Ma derived from zircon U-Pb dating of a sandy dolostone from the BACE interval in the La Ciénega Formation in Mexico also serves as a maximum age constraint on the BACE [66]. Terminal Ediacaran and possible earliest Cambrian strata in Namibia and South Africa have also been precisely dated via zircon U-Pb CA-ID-TIMS [67–69]. However, carbon isotope chemostratigraphy of these strata shows no evidence for prominent negative CIEs, which either suggests that the BACE onset postdates 538.04 ± 0.14 Ma (corresponding to the youngest dated ash bed interbedded with carbonates that record positive δ^13^C_carb_ data [68]), or is not recorded in the Nama carbonate rocks due to local effects, the evidence for which remains to be found. It is generally accepted that the BACE nadir always predates the base of the Cambrian as defined by the first appearance datum (FAD) of the ichnospecies *Treptichnus pedum* [47,65]. However, the precise age for the base of the Cambrian is not well determined, and a recent study [65] has suggested that it could be ≥5.8 Myr younger than the age of 538.8 ± 0.6 Ma used in the latest International Chronostratigraphic Chart. Thus, more high-precision dates from fossiliferous BACE-bearing Ediacaran-Cambrian transitional strata are required to calibrate the ages of the BACE and the base of the Cambrian.

### Neoproterozoic glaciations

The Neoproterozoic hosts at least two globally extensive and long-lived Snowball Earth events, the Sturtian and Marinoan, which represent global glaciations with ice sheets that extended to low latitudes for millions of years [70]. Although some pre-Sturtian and post-Marinoan glaciations are known, the majority of these were not global in extent and others remain under consideration.

The pre-Sturtian Kaigas glaciation was initially proposed based on interpreted glacial deposits of the Kaigas Formation in southern Namibia [71], the Bayisi Formation in NW China [72], the Grand Conglomerate in Zambia [73], and the Konnarock Formation in the eastern United States [74]. A rhyolite zircon U-Pb date of 752.38 ± 0.26 Ma from the overlying Rosh Pinah Formation confirms that the Kaigas Formation was deposited prior to the Sturtian glaciation [75]. However, detailed stratigraphic and tectonic mapping of the eponymous location suggests that the glacial deposits previously assigned to the Kaigas Formation instead correspond to diamictites of the Sturtian Numees Formation [75]. In NW China, SHRIMP zircon U-Pb dates of 740 ± 7 Ma and 725 ± 10 Ma are from volcanic rocks which have clear context with the Bayisi glacial deposits [72]. However, the individual zircon U-Pb dates display a substantial degree of spread, implying the possibility of inheritance and/or lead loss [75] which needs to be further tested by CA-ID-TIMS U-Pb dating. The black shale of the upper Mwashya Formation in Zambia is dated at 727.3 ± 4.9 Ma by the Re-Os isochron method, indicating that the overlying Grand Conglomerate is probably correlated to the Sturtian glaciation [76]. While the Konnarock diamictite has been precisely dated by CA-ID-TIMS zircon U-Pb at ∼752–751 Ma [74], its association with an active volcanic rift complicates the interpretation of a glaciogenic origin for the diamictites [75]. The apparent lack of synchronous glacial deposits worldwide does not presently support any Snowball Earth events during the Tonian, but it remains possible that high-latitude glacial deposits formed prior to the Sturtian glaciation in some regions.

Zircon U-Pb dating of rhyolite and ash beds from horizons below and within the Eagle Creek glacial diamictite has constrained the onset timing of the early Cryogenian glaciation in NW Canada to between 717.4 ± 0.2 and 716.9 ± 0.4 Ma [77]. Other radio-isotopic ages from strata directly underlying the early Cryogenian glacial deposits include 719.47 ± 0.29 Ma from northern Alaska [78], 719.68 ± 0.46 Ma from Ethiopia [79], and 720.2 ± 1.4 Ma from South China [80]. A zircon U-Pb date of 711.52 ± 0.20 Ma from a volcaniclastic interval within the Ghubrah diamictite provides a minimum age constraint on the onset timing of the early Cryogenian glaciation in Oman [64]. There are some volcanic ash beds within the non-glacial mid-Cryogenian Datangpo Formation, and CA-ID-TIMS U-Pb dating of zircon grains from these deposits yield precise constraints on the deglaciation timing of the early Cryogenian glaciation in South China. Here, two ash bed zircon U-Pb dates of 658.97 ± 0.22 Ma [81] and 658.80 ± 0.50 Ma [25] from strata directly overlying the Tiesi'ao diamictite in western Hunan Province indicate that the early Cryogenian glaciation probably ended prior to 659 Ma in South China. This is supported by the ash bed zircon U-Pb date of 660.98 ± 0.18 Ma from the basal Datangpo Formation in southeastern Guizhou Province where the Datangpo Formation is conformably overlying the Fulu sandstone [81]. However, the correlation between this U-Pb date and the early Cryogenian glaciation has been complicated by the uncertainty in the glaciogenic origin of the Fulu Formation [82,83], compromising the utility of this date to constrain the terminal timing of the early Cryogenian glaciation. In South Australia, an ash bed zircon U-Pb date of 663.03 ± 0.11 Ma from the uppermost Wilyerpa diamictite provides a maximum age constraint on the end of the early Cryogenian glaciation [84]. These early Cryogenian glacial deposits worldwide have been collectively regarded as the records of the Sturtian Snowball Earth event, and the age constraints discussed above support that the onset and termination of the Sturtian were globally synchronous as predicted by the Snowball Earth hypothesis.

Glacial deposits presumably corresponding to the late Cryogenian Marinoan Snowball Earth are widely distributed [70]. However, its onset timing has not been well determined by radio-isotopic dating. Available ash bed zircon U-Pb dates of 657.17 ± 0.27 Ma [81], 654.2 ± 2.7 Ma [85], and 654.5 ± 3.8 Ma [86] from the middle-upper non-glacial Datangpo Formation provide maximum age constraints on the onset of the late Cryogenian Nantuo glaciation in South China. Cyclostratigraphic estimates suggest that the duration of the Datangpo Formation is 9.8Myr [87], implying that the Nantuo glaciation could start at ∼650 Ma. A maximum depositional age of 651.69 ± 0.64 Ma from a siltstone in the upper Kingston Peak Formation of the Death Valley region, California, provides a lower age limit for the late Cryogenian Wildrose glaciation in Laurentia [51]. Recently, four ash beds within the late Cryogenian Ghaub glacial deposit were dated, and the oldest date 638.93 ± 0.32 Ma corresponds to a horizon 3.8 m above a gradational contact with the underlying Franni-aus Formation and below the first evidence for locally grounded ice, implying a maximum onset age of ∼639 Ma for hard Snowball conditions recorded within the Ghaub Formation [52]. These late Cryogenian glacial deposits have been regarded as records of the Marinoan Snowball Earth, and the available age constraints on these deposits are currently insufficient to infer the globally synchronous onset of the Marinoan Snowball Earth. The terminal timing of the Marinoan glaciation in South China has been well constrained at 635 Ma by two ash bed zircon U-Pb dates of 634.57 ± 0.88 Ma [25] and 635.23 ± 0.57 Ma [58] from the topmost diamictite and the cap carbonate, respectively. Other age constraints on the termination of the Marinoan glaciation include a zircon U-Pb date of 636.41 ± 0.34 Ma from a sandstone bed within the uppermost Cottons Breccia in Tasmania [88], an ash bed zircon U-Pb date of 635.21 ± 0.59 Ma from the upper Ghaub diamictite in Namibia [89], and a black shale Re-Os date of 632.3 ± 5.9 Ma from the post-Marinoan Sheepbed Formation in NW Canada [76]. These dates indicate that Marinoan deglaciation was globally synchronous.

There is an increasing number of glacial deposits reported from Ediacaran successions. Within them, the Gaskiers Formation and its equivalents in eastern Newfoundland have been directly dated by CA-ID-TIMS zircon U-Pb of ash beds that occur below, within and above the glacial deposits. The results indicate that the Gaskiers diamictite on the Avalon Peninsula was deposited between 580.90 ± 0.40 and 579.88 ± 0.44 Ma, and the Trinity diamictite on the Bonavista Peninsula was deposited between 579.63 ± 0.15 and 579.24 ± 0.17 Ma, resulting in a duration of less than one million years assuming approximately synchronous deglaciation [90]. A recent study has also found that the upper part of the Mall Bay Formation, which conformably underlies the Gaskiers Formation, also hosts sedimentological evidence for deposition under cold climatic conditions, including the presence of glendonite, dropstones, and iceberg-rafted debris, which together indicate that the duration of the Gaskiers glaciation is likely to have been longer than previously thought [91]. While other possible Ediacaran diamictites have been reported from nearly every palaeocontinent [92], either the age of these units is less well constrained and/or the glaciogenic origin of them remains equivocal, resulting in difficulty in assessing their synchroneity [3,93]. Nevertheless, available age constraints from detrital zircon U-Pb, chemostratigraphy, and biostratigraphy indicate that several of them were deposited in the late Ediacaran [3,92–94]. Given that the late Ediacaran witnessed the rapid rise of complex macroscopic life and the dawn of the Cambrian explosion, additional high-precision geochronological studies on the late Ediacaran glaciations are warranted to further constrain their timing, synchroneity, mechanism, and impacts on the early evolution of animals.

### Neoproterozoic fossil records

The biosphere underwent unprecedented macroevolutionary change during the Neoproterozoic. This time interval records the emergence and rapid diversification of multicellular eukaryotes and animals, which is archived in numerous exceptionally well-preserved fossil lagerstätten, including the Lantian biota and Weng'an biota of South China, and the subsequent Ediacaran fossil assemblages globally (Fig. 4), which include some of the earliest animal representatives. Precise age constraints on these records are critical for the calibration of the timescale of early animal diversification.

The Lantian biotic assemblage is preserved in black shales of the Lantian Formation, which were deposited on the lower slope to basin in southern Anhui Province, China. It constitutes a macroscopic fossil assemblage of morphologically differentiated benthic algae and putative animal affinities [95]. There are no robust radio-isotopic age constraints on the Lantian biota. The middle part of the Member II of Lantian Formation, which hosts the Lantian biota, is dated at 602 ± 7 Ma by the black shale Re-Os isochron method [8]. The upper boundary of the Lantian biota interval is near the Lantian Member II-III boundary. This horizon is concurrent with a negative CIE below the correlated DOUNCE/Shuram interval, which is interpreted as an equivalent to the one recorded near the Doushantuo Member II-III boundary in the Yangtze Gorges area. This CIE in the Yangtze Gorges area occurs near the transition between the *Tanarium conoideum-Cavaspina basiconica* and *Tanarium pycnacanthum-Ceratosphaeridium glaberosum* acritarch assemblage biozones and is correlated to the 580 Ma Gaskiers glaciation. The Lantian biota is therefore interpreted to be older than 580 Ma [8].

The Weng'an biota occurs in the upper part of the Doushantuo Formation in the Weng'an area in Guizhou Province, China, and hosts exceptionally well-preserved eukaryotic microfossils in phosphorite. Precise age constraints on the Weng'an biota are still lacking. The lowest part of the Weng'an biota interval is near the Sequence 1/2 boundary of the Doushantuo Formation and a negative CIE [2,59]. This horizon is marked by a clay bed at the Zhangcunping section which was previously dated at 609 ± 5 Ma using the SIMS zircon U-Pb method [96], but CA-ID-TIMS U-Pb analyses of zircons from this bed indicate their detrital origin and the youngest CA-ID-TIMS date 612.5 ± 0.9 Ma provides the maximum depositional age. A correlated horizon at the Jiulongwan section is dated at 587.2 ± 3.6 Ma using the black shale Re-Os isochron method [2]. The Weng'an biota extends to the uppermost part of the Doushantuo Formation, which records a DOUNCE/Shuram equivalent negative CIE. Thus, the upper limit of the Weng'an biota is probably at ∼575–570 Ma.

Globally distributed complex macroscopic fossils of the Ediacara biota have been broadly grouped into three assemblages, from oldest to youngest: the Avalon, White Sea, and Nama assemblages [97]. The Avalon assemblage is characterized by deep-water communities and has been well constrained by ash bed zircon U-Pb ages ranging from 574.17 ± 0.19 Ma to 564.13 ± 0.20 Ma in Newfoundland [37], and to between 565.22 ± 0.33 Ma and 556.6 ± 6.4 Ma (ash bed zircon LA-ICP-MS U-Pb age) in the Charnwood area, England [98]. Some representatives of the Avalon assemblage continue to be preserved in globally-distributed strata that post-date the fossiliferous Avalonian deposits of Newfoundland and central England, indicating a long temporal range for the Avalon assemblage [99]. The White Sea assemblage represents the apex of diversity and disparity among Ediacaran macrofossil deposits. Two ash deposits interbedded with strata that host White Sea assemblage macrofossils in the White Sea area of northwestern Russia, are dated at 557.28 ± 0.14 Ma and 552.96 ± 0.19 Ma [2]. The FAD of *Dickinsonia*, together with the oldest metazoan lipid biomarkers, is ∼90 m below the 557.28 Ma ash bed, and one of the oldest bilaterian body fossils, *Kimberella*, which is a probable lophotrochozoan, occurs slightly above the 557.28 Ma ash bed in the White Sea area [2]. Possible *Dickinsonia* specimens have also been reported from the Dengying Formation in South China [100]. The lower boundary of the Nama assemblage is constrained by a negative CIE, which is assumed correlative to a CIE from immediately above an ash bed dated to 550 Ma from the Yangtze Gorges area of South China [2]. The upper limits of Nama assemblage fossils are radiometrically constrained to be <538.74 Ma (tubular and skeletal cloudinids) and <538.57 Ma (soft-bodied erniettomorphs) by high-precision zircon U-Pb dates from the uppermost Schwarzrand Subgroup of the Nama Group in Namibia and northwest Republic of South Africa [67,68]. While the main phases of assemblage dominance are distinctive, there are temporal overlaps between the Avalon, White Sea, and Nama assemblages.

## NEOPROTEROZOIC EVOLUTION

The advances in high-precision age constraints on the carbonate carbon isotope excursions, large-scale magmatism, glaciations, and fossil assemblages in the Neoproterozoic permit a better understanding of the interactions between these events. For example, the interactions between the Franklin LIP and the onset of the Sturtian Snowball Earth, and between environmental change and life evolution, have been reassessed based on their refined relative temporal calibrations.

LIPs can affect climate change in multiple ways. Volcanic eruptions cause transient climate cooling through injection of sulfate aerosols into the stratosphere, and the climate is subsequently expected to return to its background state within years unless the aerosol-driven cooling effect is sufficient to result in ice-albedo runaway and resulting Snowball Earth [101]. Emplacement of LIPs could increase global weatherability and therefore cooling; however, the magnitude of LIP-driven chemical weathering is controlled by its surface extent, latitude and topography of its locality, background climate state, and the regolith and soil shielding that would have developed several million years after emplacement [102]. Statistical analyses [102] and modeling [103] suggest that most LIPs have limited significance for global cooling on long timescales. By contrast, the elevated release of volatiles via protracted outgassing and contact metamorphism with host sedimentary rocks could occur prior to, concurrent with, and subsequent to the main phase of LIP emplacement, driving climate warming and reduced biospheric stability on multimillion year timescales [104,105]. The new high-precision age constraints on the Franklin and Hubei-Shaanxi LIPs (from 720.21 ± 0.32 Ma to 718.43 ± 0.21 Ma) [106–108] and the onset of Sturtian glaciation [77] indicate that large-scale mafic magmatism occurred ∼1–3Myr prior to the onset of the Sturtian Snowball Earth. This apparent lag time may be inconsistent with direct connections between volcanic sulfate aerosol emissions and the onset of the Sturtian Snowball Earth [101]. Continued exhumation of the low-latitude Franklin and other late Tonian LIPs (Fig. 4) by superplume-induced continental doming during the break-up of the Rodinia supercontinent [109] may have substantially enhanced chemical weathering, which lowered atmospheric CO_2_ levels below the threshold required to plunge Earth into the Snowball climate state.

A recent quantitative study [110] has shown that the diversity of Neoproterozoic eukaryotes is markedly different before and after the Cryogenian glaciations (Fig. 4). Eukaryotic diversity appears to have been relatively stable in the Tonian and Cryogenian, although some minor fluctuations in diversity may have occurred in the time interval from pre- to syn-Bitter Springs CIE (Fig. 4). Redox proxies such as Cr isotopes [111] and I/(Ca + Mg) ratios [112,113] suggest that shallow marine anoxia may have been a persistent feature of Tonian paleo-environmental settings, but was interrupted by a transient atmospheric-oceanic oxygenation event during this time interval, which may be partially responsible for the concurrent fluctuations in biodiversity. However, we note that geochemical proxy data with which to constrain long term regional and global changes in Tonian paleo-environmental conditions are sparse and more data and integration into global chronostratigraphic frameworks is required.

The early Ediacaran shows an increase in total eukaryotic diversity, which is followed by a rapid decrease between the Gaskiers glaciation and the onset of DOUNCE/Shuram event (Fig. 4). From the DOUNCE/Shuram event onward, the diversity of Ediacaran eukaryotes is dominated by the macroscopic Ediacara-type organisms, which show maximum diversity during the White Sea interval and two possible extinctions, which were characterized to a very different extent based on changes to standing diversity, across the White Sea-Nama transition and Ediacaran-Cambrian transition [3,114]. At the current resolution, these transitions in the Ediacaran fossil record coincide with CIEs (Fig. 4), which are suggestive of a potential causal relationship between environmental perturbations recorded in the carbon cycle and biological turnovers [2]. Thus, glaciations and changing ocean chemistry may have exerted critical extrinsic controls on evolution, demonstrating complex interactions in the Neoproterozoic Earth-life system.

## SUMMARY AND FUTURE DIRECTIONS

The advances in total procedural blank, chemical abrasion, U-Pb tracers, mass spectrometry, and data interpretation have significantly increased the precision, accuracy, and inter-lab reproducibility of high-precision CA-ID-TIMS zircon U-Pb dating results. The continuing application of CA-ID-TIMS zircon U-Pb dating to sedimentary successions and large-scale magmatism is resulting in an increasing number of high-precision radio-isotopic age constraints with which to calibrate timings and rates of major events in deep time [115,116]. The temporally calibrated LIPs, glaciations, fossil biodiversity data, and carbonate carbon isotope profile of the Neoproterozoic present evidence for connections between Earth's interior and exosphere, and between environmental change and life evolution, providing critical insights into the coevolution of Earth and life in the Neoproterozoic Era.

Synthetic ^202^Pb and ^205^Pb tracers are the cornerstones of ID-TIMS U-Pb geochronology. All of the synthetic Pb tracers used by the ID-TIMS U-Pb community were produced in the last century. The increasing usage and limited amount of the synthetic Pb tracers make their availability the ‘Sword of Damocles’ for ID-TIMS U-Pb geochronology [117]. Thus, an urgent effort is needed to secure new sources of synthetic Pb tracers. Besides, sample-specific customization of chemical abrasion conditions and continued recalibration the U decay constants are also important directions to further advance CA-ID-TIMS zircon U-Pb geochronology.

Although there has been a marked increase in the number of radio-isotopic age constraints for Neoproterozoic sedimentary successions, some key horizons (such as the onset of the late Cryogenian glaciation, the DOUNCE/Shuram, and the basal Cambrian) lack direct high-precision age constraints, and a temporal framework of sufficient resolution is yet to be achieved for the Neoproterozoic. While dating more volcanic interbeds within Neoproterozoic successions is the most straightforward way to advance this effort, the datable materials are often intermittently distributed and are not necessarily co-located with the record of interest, which demands a fully integrated approach that considers available geochronologic and chemostratigraphic data. High-precision dating of detrital zircon grains from widely distributed siliciclastic rocks by combining SIMS/LA-ICP-MS and CA-ID-TIMS methods [51,118] provide precise maximum age constraints which, together with other direct radio-isotopic ages and Bayesian age-depth models, can refine the temporal framework of a sequence and estimate the depositional ages of key horizons. Another key to this effort is the continuation and development of new collaborations between the high-precision geochronology and astrochronology communities, which could facilitate the combination of absolute age constraints with floating astronomical time scales to produce a highly resolved Neoproterozoic geochronology.

## Supplementary Material

nwaf206_Supplemental_File

## References

[1] Park Y, Swanson-Hysell NL, MacLennan SA et al. The lead-up to the Sturtian Snowball Earth: Neoproterozoic chemostratigraphy time-calibrated by the Tambien Group of Ethiopia. GSA Bull 2020; 132: 1119–49. 10.1130/B35178.1

[2] Yang C, Rooney AD, Condon DJ et al. The tempo of Ediacaran evolution. Sci Adv 2021; 7: eabi9643. 10.1126/sciadv.abi9643 34731004 PMC8565906

[3] Bowyer FT, Wood RA, Yilales M. Sea level controls on Ediacaran-Cambrian animal radiations. Sci Adv 2024; 10: eado6462. 10.1126/sciadv.ado6462 39083611 PMC11290527

[4] Bold U, Smith EF, Rooney AD et al. Neoproterozoic stratigraphy of the Zavkhan terrane of Mongolia: the backbone for Cryogenian and early Ediacaran chemostratigraphic records. Am J Sci 2016; 315: 1–63. 10.2475/01.2016.01

[5] Yang C, Bowyer FT, Condon DJ et al. New U-Pb age from the Shuijingtuo Formation (Yangtze Gorges area) and its implications for the Cambrian timescale. Palaeogeogr Palaeoclimatol Palaeoecol 2023; 616: 111477. 10.1016/j.palaeo.2023.111477

[6] Condon DJ, Bowring SA. A user's guide to Neoproterozoic geochronology. In: Arnaud E, Halverson GP, Shields-Zhou G (eds.). The Geological Record of Neoproterozoic Glaciations. London: The Geological Society, 2011.

[7] Rooney AD, Cantine MD, Bergmann KD et al. Calibrating the coevolution of Ediacaran life and environment. Proc Natl Acad Sci USA 2020; 117: 16824–30. 10.1073/pnas.2002918117 32632000 PMC7382294

[8] Yang C, Li Y, Selby D et al. Implications for Ediacaran biological evolution from the ca. 602 Ma Lantian biota in China. Geology 2022; 50: 562–6. 10.1130/G49734.1

[9] Li Y, Glorie S, Selby D. Re-Os geochronology for sulfides and organic-rich materials. Natl Sci Rev 2025; 12: nwaf300. 10.1093/nsr/nwaf300 PMC12416286 40927435

[10] Condon D, Schoene B, Schmitz M et al. Recommendations for the reporting and interpretation of isotope dilution U-Pb geochronological information. GSA Bull 2024; 136: 4233–51. 10.1130/B37321.1

[11] Schaltegger U, Ovtcharova M, Schoene B. High-precision CA-ID-TIMS U-Pb geochronology of zircon. In: Shellnutt JG, Denyszyn SW, Suga K (eds.). Methods and Applications of Geochronology. Amsterdam: Elsevier, 2024, 19–52.

[12] Hu Z, Li X-H, Luo T et al. Tanz zircon megacrysts: a new zircon reference material for the microbeam determination of U–Pb ages and Zr–O isotopes. J Anal At Spectrom 2021; 36: 2715–34. 10.1039/D1JA00311A

[13] Markovic S, Wotzlaw J-F, Szymanowski D et al. µID-TIMS: spatially resolved high-precision U–Pb zircon geochronology. Geochronology 2024; 6: 621–38. 10.5194/gchron-6-621-2024

[14] Crowley JL . Laser cutting of zircon for CA-TIMS geochronology: adding spatial resolution to high-precision dates. Geological Society of America Abstracts with Programs 2018 Vol. 50, No. 6, Paper No. 12-2, Indianapolis.

[15] Krogh TE . Improved accuracy of U-Pb zircon ages by the creation of more concordant systems using an air abrasion technique. Geochim Cosmochim Acta 1982; 46: 637–49. 10.1016/0016-7037(82)90165-X

[16] Mattinson JM . Zircon U–Pb chemical abrasion (“CA-TIMS”) method: combined annealing and multi-step partial dissolution analysis for improved precision and accuracy of zircon ages. Chem Geol 2005; 220: 47–66. 10.1016/j.chemgeo.2005.03.011

[17] McKanna AJ, Koran I, Schoene B et al. Chemical abrasion: the mechanics of zircon dissolution. Geochronology 2023; 5: 127–51. 10.5194/gchron-5-127-2023

[18] Huyskens MH, Zink S, Amelin Y. Evaluation of temperature-time conditions for the chemical abrasion treatment of single zircons for U–Pb geochronology. Chem Geol 2016; 438: 25–35. 10.1016/j.chemgeo.2016.05.013

[19] Widmann P, Davies JHFL, Schaltegger U. Calibrating chemical abrasion: its effects on zircon crystal structure, chemical composition and U Pb age. Chem Geol 2019; 511: 1–10. 10.1016/j.chemgeo.2019.02.026

[20] McKanna AJ, Schoene B, Szymanowski D. Geochronological and geochemical effects of zircon chemical abrasion: insights from single-crystal stepwise dissolution experiments. Geochronology 2024; 6: 1–20. 10.5194/gchron-6-1-2024

[21] Parrish RR, Krogh TE. Synthesis and purification of ^205^Pb for U-Pb geochronology. Chem Geol 1987; 66: 103–10. 10.1016/0168-9622(87)90033-9

[22] Condon DJ, Schoene B, McLean NM et al. Metrology and traceability of U–Pb isotope dilution geochronology (EARTHTIME Tracer Calibration Part I). Geochim Cosmochim Acta 2015; 164: 464–80. 10.1016/j.gca.2015.05.026

[23] McLean NM, Condon DJ, Schoene B et al. Evaluating uncertainties in the calibration of isotopic reference materials and multi-element isotopic tracers (EARTHTIME Tracer Calibration Part II). Geochim Cosmochim Acta 2015; 164: 481–501. 10.1016/j.gca.2015.02.040

[24] Hiess J, Condon DJ, McLean N et al. ^238^U/^235^U systematics in terrestrial uranium-bearing minerals. Science 2012; 335: 1610–4. 10.1126/science.1215507 22461608

[25] Zhou C, Huyskens MH, Lang X et al. Calibrating the terminations of Cryogenian global glaciations. Geology 2019; 47: 251–4. 10.1130/G45719.1

[26] Schaltegger U, Ovtcharova M, Gaynor SP et al. Long-term repeatability and interlaboratory reproducibility of high-precision ID-TIMS U–Pb geochronology. J Anal At Spectrom 2021; 36: 1466–77. 10.1039/D1JA00116G 34276120 PMC8262554

[27] Szymanowski D, Schoene B. U-Pb ID-TIMS geochronology using ATONA amplifiers. J Anal At Spectrom 2020; 35: 1207–16. 10.1039/D0JA00135J

[28] Von Quadt A, Wotzlaw JF, Buret Y et al. High-precision zircon U/Pb geochronology by ID-TIMS using new 10^13^ ohm resistors. J Anal At Spectrom 2016; 31: 658–65. 10.1039/C5JA00457H

[29] Wotzlaw JF, Buret Y, Large SJE et al. ID-TIMS U-Pb geochronology at the 0.1‰ level using 10^13^ Ω resistors and simultaneous U and ^18^O/^16^O isotope ratio determination for accurate UO_2_ interference correction. J Anal At Spectrom 2017; 32: 579–86. 10.1039/C6JA00278A

[30] Bouman C, Trinquier A, Lloyd N et al. New Design 10^13^ Ω Amplifiers for Measurement of Small Ion Beam Currents. Thermo Fisher Scientific Application note 30282. https://assets.thermofisher.cn/TFS-Assets/CMD/Application-Notes/AN-30282-Triton-Plus-AN30282-EN.pdf (12 June 2025, date last accessed).

[31] Cox SE, Hemming SR, Tootell D. The Isotopx NGX and ATONA Faraday amplifiers. Geochronology 2020; 2: 231–43. 10.5194/gchron-2-231-2020

[32] Hockley M, Palacz Z, Yardley S et al. Ultra Low Noise and Baseline Drift Zeptona Faraday Detector. IsotopX Technical Note 2102. https://www.isotopx.com/resources/ultra-low-noise-and-baseline-drift-zeptona-faraday-detector (12 June 2025, date last accessed).

[33] Wang T, Ramezani J, Yang C et al. High-resolution geochronology of sedimentary strata by U-Pb CA-ID-TIMS zircon geochronology: a review. Earth-Sci Rev 2023; 245: 104550. 10.1016/j.earscirev.2023.104550

[34] Samperton KM, Schoene B, Cottle JM et al. Magma emplacement, differentiation and cooling in the middle crust: integrated zircon geochronological-geochemical constraints from the Bergell Intrusion, Central Alps. Chem Geol 2015; 417: 322–40. 10.1016/j.chemgeo.2015.10.024

[35] Keller CB, Schoene B, Samperton KM. A stochastic sampling approach to zircon eruption age interpretation. Geochem Perspect Lett 2018; 8: 31–5. 10.7185/geochemlet.1826

[36] Wotzlaw J-F, Schaltegger U, Frick DA et al. Tracking the evolution of large-volume silicic magma reservoirs from assembly to supereruption. Geology 2013; 41: 867–70. 10.1130/G34366.1

[37] Matthews JJ, Liu AG, Yang C et al. A chronostratigraphic framework for the rise of the Ediacaran macrobiota: new constraints from Mistaken Point Ecological Reserve, Newfoundland. GSA Bull 2021; 133: 612–24. 10.1130/B35646.1

[38] Schmitz MD, Kuiper KF. High-precision geochronology. Elements 2013; 9: 25–30. 10.2113/gselements.9.1.25

[39] Szymanowski D, Wotzlaw J-F, Ovtcharova M et al. Interlaboratory reproducibility of ID-TIMS U–Pb geochronology evaluated with a pre-spiked natural zircon solution. EGUsphere [preprint], doi: 10.5194/egusphere-2025-1001.

[40] Zhao W, Li Q, Liu Y et al. Long-term reproducibility of SIMS zircon U-Pb geochronology. J Earth Sci 2022; 33: 17–24. 10.1007/s12583-021-1549-1

[41] Horstwood MSA, Košler J, Gehrels G et al. Community-derived standards for LA-ICP-MS U-(Th-)Pb geochronology—uncertainty propagation, age interpretation and data reporting. Geostand Geoanal Res 2016; 40: 311–32. 10.1111/j.1751-908X.2016.00379.x

[42] Roberts NMW, Drost K, Horstwood MSA et al. Laser ablation inductively coupled plasma mass spectrometry (LA-ICP-MS) U–Pb carbonate geochronology: strategies, progress, and limitations. Geochronology 2020; 2: 33–61. 10.5194/gchron-2-33-2020

[43] Li H, Zhang S, Han J et al. Astrochronologic calibration of the Shuram carbon isotope excursion with new data from South China. Glob Planet Change 2022; 209: 103749. 10.1016/j.gloplacha.2022.103749

[44] Geyman EC, Maloof AC. A diurnal carbon engine explains ^13^C-enriched carbonates without increasing the global production of oxygen. Proc Natl Acad Sci USA 2019; 116: 24433–9. 10.1073/pnas.1908783116 31704769 PMC6900632

[45] Cui H, Warren LV, Uhlein GJ et al. Global or regional? Constraining the origins of the middle Bambuí carbon cycle anomaly in Brazil. Precambrian Res 2020; 348: 105861. 10.1016/j.precamres.2020.105861

[46] Ahm ASC, Maloof AC, Macdonald FA et al. An early diagenetic deglacial origin for basal Ediacaran “cap dolostones”. Earth Planet Sci Lett 2019; 506: 292–307. 10.1016/j.epsl.2018.10.046

[47] Bowyer FT, Zhuravlev AY, Wood R et al. Calibrating the temporal and spatial dynamics of the Ediacaran—Cambrian radiation of animals. Earth-Sci Rev 2022; 225: 103913. 10.1016/j.earscirev.2021.103913

[48] Halverson GP, Shen C, Davies JHFL et al. A Bayesian approach to inferring depositional ages applied to a late Tonian reference section in Svalbard. Front Earth Sci 2022; 10: 798739. 10.3389/feart.2022.798739

[49] Yang C, Zhu M, Condon DJ et al. Geochronological constraints on stratigraphic correlation and oceanic oxygenation in Ediacaran-Cambrian transition in South China. J Asian Earth Sci 2017; 140: 75–81. 10.1016/j.jseaes.2017.03.017

[50] Millikin AEG, Strauss JV, Halverson GP et al. Calibrating the Russøya excursion in Svalbard, Norway, and implications for Neoproterozoic chronology. Geology 2022; 50: 506–10. 10.1130/G49593.1

[51] Nelson LL, Smith EF, Hodgin EB et al. Geochronological constraints on Neoproterozoic rifting and onset of the Marinoan glaciation from the Kingston Peak Formation in Death Valley, California (USA). Geology 2020; 48: 1083–7. 10.1130/G47668.1

[52] Tasistro-Hart AR, Macdonald FA, Crowley JL et al. Four-million-year Marinoan snowball shows multiple routes to deglaciation. Proc Natl Acad Sci USA 2025; 122: e2418281122. 10.1073/pnas.2418281122 40258138 PMC12067226

[53] Hill AC, Arouri K, Gorjan P et al. Geochemistry of marine and nonmarine environments of a neoproterozoic cratonic carbonate/evaporite: the Bitter Springs Formation, Central Australia. In: Grotzinger JP, James NP (eds.). Carbonate Sedimentation and Diagenesis in the Evolving Precambrian World. Oklahoma: Society for Sedimentary Geology, 2000, 327–44.

[54] Macdonald FA, Schmitz MD, Crowley JL et al. Calibrating the Cryogenian. Science 2010; 327: 1241–3. 10.1126/science.1183325 20203045

[55] Swanson-Hysell NL, Maloof AC, Condon DJ et al. Stratigraphy and geochronology of the Tambien Group, Ethiopia: evidence for globally synchronous carbon isotope change in the Neoproterozoic. Geology 2015; 43: 323–6. 10.1130/G36347.1

[56] Cohen PA, Strauss JV, Rooney AD et al. Controlled hydroxyapatite biomineralization in an ∼810 million-year-old unicellular eukaryote. Sci Adv 2017; 3: e1700095. 10.1126/sciadv.1700095 28782008 PMC5489269

[57] Zhang Y, Zhu M. Meta-analysis of the DOUNCE event (Shuram/Wonoka excursion): pattern, variation, causal mechanism, and global correlation. Earth-Sci Rev 2025; 261: 105000. 10.1016/j.earscirev.2024.105000

[58] Condon D, Zhu M, Bowring S et al. U-Pb ages from the Neoproterozoic Doushantuo Formation, China. Science 2005; 308: 95–8. 10.1126/science.1107765 15731406

[59] Zhu M, Lu M, Zhang J et al. Carbon isotope chemostratigraphy and sedimentary facies evolution of the Ediacaran Doushantuo Formation in western Hubei, South China. Precambrian Res 2013; 225: 7–28. 10.1016/j.precamres.2011.07.019

[60] An Z, Jiang G, Tong J et al. Stratigraphic position of the Ediacaran Miaohe biota and its constrains on the age of the upper Doushantuo δ^13^C anomaly in the Yangtze Gorges area, South China. Precambrian Res 2015; 271: 243–53. 10.1016/j.precamres.2015.10.007

[61] Zhou C, Xiao S, Wang W et al. The stratigraphic complexity of the middle Ediacaran carbon isotopic record in the Yangtze Gorges area, South China, and its implications for the age and chemostratigraphic significance of the Shuram excursion. Precambrian Res 2017; 288: 23–38. 10.1016/j.precamres.2016.11.007

[62] Cantine MD, Rooney AD, Knoll AH et al. Chronology of Ediacaran sedimentary and biogeochemical shifts along eastern Gondwanan margins. Commun Earth Environ 2024; 5: 520. 10.1038/s43247-024-01630-1

[63] Zhu MY, Babcock LE, Peng SC. Advances in Cambrian stratigraphy and paleontology: integrating correlation techniques, paleobiology, taphonomy and paleoenvironmental reconstruction. Palaeoworld 2006; 15: 217–22. 10.1016/j.palwor.2006.10.016

[64] Bowring SA, Grotzinger JP, Condon DJ et al. Geochronologic constraints on the chronostratigraphic framework of the Neoproterozoic Huqf Supergroup, Sultanate of Oman. Am J Sci 2007; 307: 1097–145. 10.2475/10.2007.01

[65] Nelson LL, Crowley JL, Smith EF et al. Cambrian explosion condensed: high-precision geochronology of the lower Wood Canyon Formation, Nevada. Proc Natl Acad Sci USA 2023; 120: e2301478120. 10.1073/pnas.2301478120 37459545 PMC10372641

[66] Hodgin EB, Nelson LL, Wall CJ et al. A link between rift-related volcanism and end-Ediacaran extinction? Integrated chemostratigraphy, biostratigraphy, and U-Pb geochronology from Sonora, Mexico. Geology 2021; 49: 115–9. 10.1130/G47972.1

[67] Linnemann U, Ovtcharova M, Schaltegger U et al. New high-resolution age data from the Ediacaran–Cambrian boundary indicate rapid, ecologically driven onset of the Cambrian explosion. Terra Nova 2019; 31: 49–58. 10.1111/ter.12368

[68] Nelson LL, Ramezani J, Almond JE et al. Pushing the boundary: a calibrated Ediacaran-Cambrian stratigraphic record from the Nama Group in northwestern Republic of South Africa. Earth Planet Sci Lett 2022; 580: 117396. 10.1016/j.epsl.2022.117396

[69] Bowyer FT, Messori F, Wood R et al. Foundational uncertainties in terminal Ediacaran chronostratigraphy revealed by high-precision zircon U-Pb geochronology of the Nama Group, Namibia. Earth-Sci Rev 2025; 268: 105169. 10.1016/j.earscirev.2025.105169

[70] Hoffman PF, Abbot DS, Ashkenazy Y et al. Snowball Earth climate dynamics and Cryogenian geology-geobiology. Sci Adv 2017; 3: e1600983. 10.1126/sciadv.1600983 29134193 PMC5677351

[71] Frimmel HE, Klötzli US, Siegfried PR. New Pb-Pb single zircon age constraints on the timing of Neoproterozoic glaciation and continental break-up in Namibia. J Geol 1996; 104: 459–69. 10.1086/629839

[72] Xu B, Xiao S, Zou H et al. SHRIMP zircon U-Pb age constraints on Neoproterozoic Quruqtagh diamictites in NW China. Precambrian Res 2009; 168: 247–58. 10.1016/j.precamres.2008.10.008

[73] Key RM, Liyungu AK, Njamu FM et al. The western arm of the Lufilian Arc in NW Zambia and its potential for copper mineralization. J Afr Earth Sci 2001; 33: 503–28. 10.1016/S0899-5362(01)00098-7

[74] MacLennan SA, Eddy MP, Merschat AJ et al. Geologic evidence for an icehouse Earth before the Sturtian global glaciation. Sci Adv 2020; 6: eaay6647. 10.1126/sciadv.aay6647 32577504 PMC7286673

[75] Pu JP, Macdonald FA, Smith EF et al. Tonian basins record rifting of Kalahari from Rodinia and no evidence of a pre-Sturtian Kaigas glaciation. Earth Planet Sci Lett 2023; 624: 118472. 10.1016/j.epsl.2023.118472

[76] Rooney AD, Strauss JV, Brandon AD et al. A Cryogenian chronology: two long-lasting synchronous Neoproterozoic glaciations. Geology 2015; 43: 459–62. 10.1130/G36511.1

[77] Macdonald FA, Schmitz MD, Strauss JV et al. Cryogenian of Yukon. Precambrian Res 2018; 319: 114–43. 10.1016/j.precamres.2017.08.015

[78] Cox GM, Strauss JV, Halverson GP et al. Kikiktat volcanics of Arctic Alaska—melting of harzburgitic mantle associated with the Franklin large igneous province. Lithosphere 2015; 7: 275–95. 10.1130/L435.1

[79] MacLennan S, Park Y, Swanson-Hysell N et al. The arc of the Snowball: U-Pb dates constrain the Islay anomaly and the initiation of the Sturtian glaciation. Geology 2018; 46: 539–42. 10.1130/G40171.1

[80] Lan Z, Huyskens MH, Lu K et al. Toward refining the onset age of Sturtian glaciation in South China. Precambrian Res 2020; 338: 105555. 10.1016/j.precamres.2019.105555

[81] Rooney AD, Yang C, Condon DJ et al. U-Pb and Re-Os geochronology tracks stratigraphic condensation in the Sturtian snowball Earth aftermath. Geology 2020; 48: 625–9. 10.1130/G47246.1

[82] Zhu M, Zhang J, Yang A et al. Neoproterozoic stratigraphy, depositional environments and hydrocarbon source-reservoir-seal bed assemblage in South China. In: Wang T (ed.). Meso-Neoproterozoic Geology and Petroleum Resources in China. Beijing: Science Press; Singapore: Springer, 2022, 181–227.

[83] Wu C-Z, Zhao F-F, Yang T et al. Genesis of the Fulu Cryogenian iron formation in South China: synglacial or interglacial? Precambrian Res 2022; 376: 106689. 10.1016/j.precamres.2022.106689

[84] Cox GM, Isakson V, Hoffman PF et al. South Australian U-Pb zircon (CA-ID-TIMS) age supports globally synchronous Sturtian deglaciation. Precambrian Res 2018; 315: 257–63. 10.1016/j.precamres.2018.07.007

[85] Liu P, Li X, Chen S et al. New SIMS U–Pb zircon age and its constraint on the beginning of the Nantuo glaciation. Sci Bull 2015; 60: 958–63. 10.1007/s11434-015-0790-3

[86] Zhang S, Jiang G, Han Y. The age of the Nantuo formation and Nantuo glaciation in South China. Terra Nova 2008; 20: 289–94. 10.1111/j.1365-3121.2008.00819.x

[87] Bao X, Zhang S, Jiang G et al. Cyclostratigraphic constraints on the duration of the Datangpo Formation and the onset age of the Nantuo (Marinoan) glaciation in South China. Earth Planet Sci Lett 2018; 483: 52–63. 10.1016/j.epsl.2017.12.001

[88] Calver CR, Crowley JL, Wingate MTD et al. Globally synchronous Marinoan deglaciation indicated by U-Pb geochronology of the Cottons Breccia, Tasmania, Australia. Geology 2013; 41: 1127–30. 10.1130/G34568.1

[89] Prave AR, Condon DJ, Hoffmann KH et al. Duration and nature of the end-Cryogenian (Marinoan) glaciation. Geology 2016; 44: 631–4. 10.1130/G38089.1

[90] Pu JP, Bowring SA, Ramezani J et al. Dodging snowballs: geochronology of the Gaskiers glaciation and the first appearance of the Ediacaran biota. Geology 2016; 44: 955–8. 10.1130/G38284.1

[91] Fitzgerald DM, Narbonne GM, Pufahl PK et al. The Mall Bay Formation (Ediacaran) and the protracted onset of the Gaskiers glaciation in Newfoundland, Canada. Precambrian Res 2024; 405: 107369. 10.1016/j.precamres.2024.107369

[92] Wang R, Shen B, Lang X et al. A great late Ediacaran ice age. Natl Sci Rev 2023; 10: nwad117. 10.1093/nsr/nwad117 37389143 PMC10306365

[93] Wong Hearing T, Tindal B, Vandyk T et al. Ediacaran coupling of climate and biosphere dynamics. EarthArXiv [Preprint], doi: 10.31223/X5S42P.

[94] Sun L, Khan MMSS, Yang C et al. Cryogenian and Ediacaran integrative stratigraphy, biotas, and paleogeographical evolution of the Qinghai-Tibetan Plateau and its surrounding areas. Sci China Earth Sci 2024; 67: 919–49. 10.1007/s11430-023-1228-x

[95] Yuan X, Chen Z, Xiao S et al. An early Ediacaran assemblage of macroscopic and morphologically differentiated eukaryotes. Nature 2011; 470: 390–3. 10.1038/nature09810 21331041

[96] Zhou C, Li X-H, Xiao S et al. A new SIMS zircon U–Pb date from the Ediacaran Doushantuo Formation: age constraint on the Weng'an biota. Geol Mag 2017; 154: 1193–201. 10.1017/S0016756816001175

[97] Waggoner B . The Ediacaran biotas in space and time. Integr Comp Biol 2003; 43: 104–13. 10.1093/icb/43.1.104 21680415

[98] Noble SR, Condon DJ, Carney JN et al. U-Pb geochronology and global context of the Charnian supergroup, UK: constraints on the age of key Ediacaran fossil assemblages. GSA Bull 2015; 127: 250–65. 10.1130/B31013.1

[99] Grazhdankin DV, Balthasar U, Nagovitsin KE et al. Carbonate-hosted Avalon-type fossils in Arctic Siberia. Geology 2008; 36: 803–6. 10.1130/G24946A.1

[100] Wang X-P, Chen Z, Pang K et al. Dickinsonia from the Ediacaran Dengying Formation in the Yangtze Gorges area, South China. Palaeoworld 2021; 30: 602–9. 10.1016/j.palwor.2021.01.002

[101] Macdonald FA, Wordsworth R. Initiation of Snowball Earth with volcanic sulfur aerosol emissions. Geophys Res Lett 2017; 44: 1938–46. 10.1002/2016GL072335

[102] Park Y, Swanson-Hysell NL, Lisiecki LE et al. Evaluating the relationship between the area and latitude of large igneous provinces and Earth's long-term climate state. In: Ernst RE, Dickson AJ, Bekker A (eds.). Large Igneous Provinces: A Driver of Global Environmental and Biotic Changes. Hoboken: Wiley, 2021, 153–68.

[103] Longman J, Mills BJW, Merdith AS. Limited long-term cooling effects of Pangaean flood basalt weathering. Nat Commun 2025; 16: 4813. 10.31223/X5HQ3F 40410180 PMC12102204

[104] Tian X, Buck WR. Intrusions induce global warming before continental flood basalt volcanism. Nat Geosci 2022; 15: 417–22. 10.1038/s41561-022-00939-w

[105] Black BA, Karlstrom L, Mills BJW et al. Cryptic degassing and protracted greenhouse climates after flood basalt events. Nat Geosci 2024; 17: 1162–8. 10.1038/s41561-024-01574-3

[106] Lu K, Mitchell RN, Yang C et al. Widespread magmatic provinces at the onset of the Sturtian snowball Earth. Earth Planet Sci Lett 2022; 594: 117736. 10.1016/j.epsl.2022.117736

[107] Pu JP, Macdonald FA, Schmitz MD et al. Emplacement of the Franklin large igneous province and initiation of the Sturtian Snowball Earth. Sci Adv 2022; 8: eadc9430. 10.1126/sciadv.adc9430 36417531 PMC9683727

[108] Dufour F, Davies JHFL, Greenman JW et al. New U-Pb CA-ID TIMS zircon ages implicate the Franklin LIP as the proximal trigger for the Sturtian Snowball Earth event. Earth Planet Sci Lett 2023; 618: 118259. 10.1016/j.epsl.2023.118259

[109] Li Z-X, Liu Y, Ernst R. A dynamic 2000—540 ma Earth history: from cratonic amalgamation to the age of supercontinent cycle. Earth-Sci Rev 2023; 238: 104336. 10.1016/j.earscirev.2023.104336

[110] Tang Q, Zheng W, Zhang S et al. Quantifying the global biodiversity of Proterozoic eukaryotes. Science 2024; 386: eadm9137. 10.1126/science.adm9137 39700282

[111] Planavsky NJ, Reinhard CT, Wang X et al. Low Mid-Proterozoic atmospheric oxygen levels and the delayed rise of animals. Science 2014; 346: 635–8. 10.1126/science.1258410 25359975

[112] Lu W, Wörndle S, Halverson GP et al. Iodine proxy evidence for increased ocean oxygenation during the Bitter Springs Anomaly. Geochem Perspect Lett 2017; 5: 53–7. 10.7185/geochemlet.1746

[113] Wörndle S, Crockford PW, Kunzmann M et al. Linking the Bitter Springs carbon isotope anomaly and early Neoproterozoic oxygenation through I/[Ca + Mg] ratios. Chem Geol 2019; 524: 119–35. 10.1016/j.chemgeo.2019.06.015

[114] Boag TH, Darroch SAF, Laflamme M. Ediacaran distributions in space and time: testing assemblage concepts of earliest macroscopic body fossils. Paleobiology 2016; 42: 574–94. 10.1017/pab.2016.20

[115] Schoene B, Eddy MP, Samperton KM et al. U-Pb constraints on pulsed eruption of the Deccan Traps across the end-Cretaceous mass extinction. Science 2019; 363: 862–6. 10.1126/science.aau2422 30792300

[116] Zhang Z, Yang C, Sahy D et al. Tempo of the late Ordovician mass extinction controlled by the rate of climate change. Sci Adv 2025; 11: eadv6788. 10.1126/sciadv.adv6788 40446039 PMC12124363

[117] Ickert RB, Eddy MP. The spike of Damocles: availability of ^205^Pb and ^202^Pb threatens the future of U-Pb and Pb-Pb ID geochronology. 2024 Goldschmidt Conference. Chicago, 18–23 August 2024.

[118] Yang C, Li X-H, Zhu M et al. Geochronological constraint on the Cambrian Chengjiang biota, South China. J Geol Soc London 2018; 175: 659–66. 10.1144/jgs2017-103

[119] Black LP, Kamo SL, Allen CM et al. Improved ^206^Pb/^238^U microprobe geochronology by the monitoring of a trace-element-related matrix effect; SHRIMP, ID–TIMS, ELA–ICP–MS and oxygen isotope documentation for a series of zircon standards. Chem Geol 2004; 205: 115–40. 10.1016/j.chemgeo.2004.01.003

[120] Bowyer FT, Krause AJ, Song Y et al. Biological diversification linked to environmental stabilization following the Sturtian Snowball glaciation. Sci Adv 2023; 9: eadf9999. 10.1126/sciadv.adf9999 37624887 PMC10456883

[121] Ernst RE, Bond DPG, Zhang S et al. Large igneous province record through time and implications for secular environmental changes and geological time-scale boundaries. In: Ernst RE, Dickson AJ, Bekker A (eds.). Large Igneous Provinces: A Driver of Global Environmental and Biotic Changes. Hoboken: Wiley, 2021, 1–26.

